# Variables Predicting Psychological Help Seeking Attitudes: Self-Stigma, Mental Health Literacy, and Depression/Anxiety

**DOI:** 10.1192/j.eurpsy.2024.1409

**Published:** 2024-08-27

**Authors:** H. Gündoğdu, K. Aslan, H. C. Ayhan, C. M. Aktaş

**Affiliations:** psychiatric and mental health nursing, Van Yüzüncü Yıl University, van, Türkiye

## Abstract

**Introduction:**

Psychological help-seeking attitudes are influenced by various factors, including self-stigma, mental health literacy, and levels of depression and anxiety. Several studies have examined the relationships between these variables and help-seeking attitudes, shedding light on the predictors of seeking psychological help. Self-stigma, or the internalization of negative attitudes towards seeking psychological help, has been found to significantly impact help-seeking attitudes. Individuals who experience higher levels of self-stigma are more likely to hold negative attitudes towards seeking help. Additionally, mental health literacy, which refers to knowledge and understanding of mental health issues, has been identified as another important predictor of help-seeking attitudes. Individuals with higher mental health literacy are more likely to have positive attitudes towards seeking psychological help. Depression and anxiety, two common mental health concerns, have also been found to influence help-seeking attitudes. Research has shown that individuals with higher levels of depression and anxiety symptoms are more likely to express intentions to seek counseling for psychological and interpersonal concerns. These mental health concerns can serve as motivators for individuals to seek professional help. Furthermore, cultural and demographic factors can also play a role in shaping help-seeking attitudes.

**Objectives:**

This study was planned to examine the variables predicting psychological help seeking attitudes: self-stigma, mental health literacy, and depression/anxiety

**Methods:**

The sample of the study was determined by purposive sampling method. The study was conducted with individuals who willing to participate the study and above 18 years age.

Individuals who saw the online advertisement and click on the study’s link were be brought to the study’s home page on Online Surveys. Should they wish to proceed, they will be brought to an information page detailing the purpose of the study, how their confidentiality and anonymity will be preserved and how their data will be treated.

Socio-Demographic Data Form, Mental Health Literacy Scale, Self-Stigma of Seeking Psychological Help Scale and Attitudes Towards Seeking Psychological Help Scale were used. Data analyses was planned to run via Statistical Package for the Social Sciences version, 27.0.

**Results:**

The analysis of the data is still ongoing in detail by the researchers. The findings and relational implications of the study will be presented.

**Conclusions:**

In conclusion, self-stigma, mental health literacy, and levels of depression and anxiety are important variables that predict psychological help-seeking attitudes. Understanding these factors can inform the development of interventions and strategies to promote help-seeking behaviors and reduce barriers to seeking psychological help.

**Disclosure of Interest:**

None Declared

